# Strategies of the War on Cancer: To Kill or to Neutralize?

**DOI:** 10.3389/fonc.2018.00667

**Published:** 2019-01-10

**Authors:** Anatoly V. Lichtenstein

**Affiliations:** Institute of Carcinogenesis, N.N. Blokhin Cancer Research Center, Moscow, Russia

**Keywords:** war on cancer, hallmarks of cancer, killer function, tumor/host relationships, cancer therapy, treatment of cancer

## Introduction

Fifteen years ago, the question “Why do we lose the war on cancer?” sounded (1). After an interview with the world's leading oncologists, the author presented, in particular, explanations such as: “Cancer is a challenge like no other,” “A very tough set of problems,” and “Focus on individual cellular mechanisms to the near exclusion of what's happening in the organism as a whole.” One of the solutions to the problem would be “Changing the way we think about cancer.”

Until now, this radical turn has not occurred: the global cancer incidence is growing and many problems remain unsolved. As a result, the advice to change the way we think about cancer and cancer treatment has become even more relevant.

## Changing the Way We Think About Cancer

Cancer is usually viewed as a disease of old age, alongside diseases such as myocardial infarction, stroke, diabetes, Parkinson's, and Alzheimer's. However, there is a radical difference between cancer and these other diseases (2). All other diseases of old age arise due to loss of a specific function, for whatever reason, and loss of specific cells, whereas cancer is a gain of a new function and new specific cells. At the dawn of oncology, solid tumors were viewed as chaotic cell conglomerates, but currently a tumor is viewed as an organ (3–5), i.e., “an anatomically discrete collection of tissues integrated to perform specific functions” (3). The fact that, with very few exceptions (6), cancer is encountered in all animals (7, 8) suggests the great evolutionary significance of this organ. However, contrary to the general practice of biological and medical research, most cancer studies are performed without any connection to this organ's origin and function.

The function of the cancer organ is to kill the organism. Although this statement seems counter-intuitive, it is actually stating an obvious fact (2, 4, 9). As was suggested by Sommer, “cancer kills the individual and saves the species” (10), and this assignment determines its important role in evolution (8). Cancer-associated genes are essential; their mutant forms are harmful to fitness, and they are therefore subjected to strong negative selection (11). A computer simulation found that additional protections against cancer increase genetic predisposition to disease in the population (12). It was hypothesized that, in evolutionary terms, cancer serves as a guardian of the gene pool (2) or a quality control mechanism (13).

Many researchers do not share this view, and instead believe that cancer is selectively neutral, since most people whom it kills are in the postreproductive age (8). This objection seems not to be convincing. In reality, cancer always arises in an organism that is genetically compromised to some extent. Usually, driver mutations in the germinal cells and zygote induce cancer during the patient's reproductive age (the early-life cancer), whereas postzygotic mutations accumulating in somatic stem cells lead to progressive genetic mosaicism and, as a result, the postreproductive late-life cancer (14–19). Most likely, it is the early-life cancer that is subject to negative selection, thus preventing the spread of mutant alleles in the population.

However, this raises the question of why the late-life cancer incidence is so high. Perhaps the overwhelming, though selectively neutral, late-life cancer is accounted for by the phenomenon of antagonistic pleiotropy (20). It has been suggested that a beneficial early-life program can continue to function at an old age (even if useless and harmful) merely “by inertia,” i.e., due to the inefficiency of evolutionary mechanisms at a postreproductive age (4). Thus, the harmful late-life cancer may be an evolutionary tradeoff for the beneficial early-life cancer. In addition, aging-related disorders of angiogenesis, metabolism, and apoptosis create favorable conditions for cancer progression (21).

It seems likely that, in the wild, where this phenomenon has evolved over millions of years, the proportion of older individuals in the population is small (as most perish due to hunger, cold, injuries, diseases, and predators), and the selectively active early-life cancer dominates. In contrast, the current human population has overcome the calamities of the wild and achieved an unprecedented increase in life expectancy (22), and the proportion of older individuals is significant. In this situation, the selectively active inherited and familial cancers that comprise < 10% of all cancer cases (23) are overshadowed by the many-fold greater numbers of selectively neutral sporadic late-life cancers. The statistics of cancer incidence of only one, far from typical, representative of the animal world (*Homo sapiens*) are able to obscure the evolutionary role of cancer in general. In this manner, cancer probably performs at the population level the same function that apoptosis does at the cellular level (24, 25).

## Changing the Way We Think About Cancer Treatment

More important, however, is the practical side of this issue. In the current framework, several hallmarks of cancer are known, which are allegedly necessary and sufficient for the formation of a tumor (26, 27). Together, they explain the development of a tumor, but not that of a *malignant* tumor. Indeed, cancer death is, in general, due to systemic damages rather than local effects of the primary tumor growth or even the metastases (5). A well-known list of hallmarks seems to reflect a predominantly *in vitro*-centric view of cancer, whereas it leaves a conceptual gap between the cancer cell phenotype and the cancer disease in the *in vivo*-centric perception of cancer, i.e., between the cancer hallmarks and multiple paraneoplastic syndromes (28–30). It is the latter, and not the metastases themselves, that are usually fatal. Hence, we have to admit that the known hallmarks of cancer, most of which are also inherent in benign tumors (31), are necessary but insufficient to completely describe the oncological process.

There is an opinion that an individual cancer cell carries no recognizable molecules or structures that make it consistently distinguishable from a normal cell (32). However, it should be recognized that the unique and extremely important hallmark of a cancer cell is its ability to systemically ruin the body (2, 4, 9). It is strange enough that such an obvious ability (in essence, a killer function) has not yet acquired a specific name of its own. There are local and generalized manifestations of tumor growth; their relative contribution varies depending on the tumor type and location. A growing tumor can affect the body locally by squeezing the brain, squeezing the vessels, causing bleeding, or promoting obturation or perforation of a hollow organ. These local manifestations are relatively well treated with surgery or irradiation, whereas the systemic effects of a tumor are not always amenable to treatment and are the cause of death of the majority of cancer patients (cancer cell dissemination makes their elimination unfeasible and a lethal outcome often unavoidable).

The deadly effects of the “incognito” function are realized through the cancer secretome (33–36), which includes extracellular vesicles (37–42), miRNAs (43), free DNA (44), and cancer-related neurogenetic factors (45). With their help, the tumor forms its own and premetastatic niches (46–48); provides itself with a blood supply (49), energy supply (50), and innervation (51); controls the microenvironment; and recruits normal cells. In the same way, a tumor induces systemic changes, such as anemia (52), coagulopathy (53), chronic inflammation (54), metabolic abnormalities (55), anorexia, cachexia (56, 57), and NETosis (58, 59). Individual components contributing to the systemic effects are expressed to varying degrees in different forms of cancer, but on the whole, the oncologic process develops as a programmed and coordinated attack of many specialized cells (including non-transformed cells) that disturbs the organismal integrity. Collectively, these data suggest that it is the special killer function that makes the tumor malignant.

The primary goal in oncology has been, and still is, to efficiently and effectively kill cancer cells (60). If we imagine, however, using this strategy to combat external human enemies (poisonous animals, for example), it becomes clear that it is fundamentally flawed. First, the task of total extermination is hardly feasible in practice; second, the off-target damages are practically unavoidable; and, finally, it is fraught with disastrous consequences for humanity due to unacceptable ecological imbalances. Nevertheless, this is the strategy currently being used in the war against cancer, for want of a better one, and cancer patients face precisely these consequences. The low efficacy and side effects of today's cancer therapy offer grounds for criticism and require alternative solutions (61–67).

In the fight against external enemies (poisonous animals), we apply a much more effective and less devastating strategy of neutralization of poisons by specific antidotes. There are a number of interesting examples of using a similar anticancer strategy in experimental mouse models: (i) oral administration of soda solution neutralizes intratumor acidity and suppresses tumor growth (68, 69); (ii) antibodies to interleukin-23 raise the immune response (70); (iii) sympathectomy blocks prostate tumor development at an early stage, and a blockade of cholinergic receptors blocks metastasis (51) [of note, an epidemiologic study indicated a decrease in mortality in prostate cancer patients receiving beta-blockers (71)]; (iv) administration of DNase I dissolves NETs, which have a high DNA content, and restores perfusion in the kidney and heart (58, 59); and (v) the TLR7/8/9 antagonist IMO-8503 inhibits cancer-induced cachexia (72). However, no matter how promising these studies may be, they are rather fragmented.

Obviously, the possibility of developing an effective antidote is determined, first of all, by the knowledge of the mechanism of poison action. Unfortunately, we still do not have a clear understanding of the disturbances the tumor introduces into the host ecosystem, without which restoration of normal homeostasis is hardly possible. Key questions remain unanswered: What is the mechanism of malignancy? Is it the same for different types of cancer (and are paraneoplastic syndromes its side effects)? Or, on the contrary, is the mechanism specific for each form of cancer? What is the relative contribution of various components of the cancer secretome? Which metabolic pathways, and in which tissues, are their key targets?

The main maladies of contemporary mankind, aging and cancer, are both deeply rooted in the biology of multicellular organisms. This explains why fighting both is so difficult. Their study requires a systemic approach integrating the molecular, cellular, tissue, and organism levels. This opportunity has appeared relatively recently and is actively used in relation to aging. New high-throughput “omics” technologies (genomics, metagenomics, methylomics, transcriptomics, metabolomics, proteomics, etc.) are now revealing information about biological pathways that change with age in different organs and tissues (73). As for cancer, the research focuses, in general, on the tumor itself and its microenvironment. There are no attempts to apply the “omics” technologies to the tumor-bearing organism for investigation of biological pathways that change in different organs and tissues with tumor progression. Supplementing the reductionist methods traditionally used in cancer research with a holistic approach that can provide vital information for disease prevention and treatment is urgently needed.

As for the identification of putative “poisons” (i.e., mediators of the killer function), one can suggest the high utility of the mouse model of parabiosis that more than 30 years ago gave evidence of the humoral mediation of cancer-associated cachexia (74, 75). This model has a number of important advantages: (i) isolation, in short-term experiments, of only systemic effects (eliminating local effects); (ii) avoidance of the chronic effects of tumor growth fraught with various side effects; and (iii) the possibility to identify active humoral factors and their immediate targets. This model has improved in recent years to provide important information concerning carcinogenesis (76–80).

## Conclusion

In parallel with the evolution of concepts on the nature of cancer, a rethinking of the tumor/host relationship has occurred. Early ideas about their antagonism and the life-and-death struggle have been exchanged for the realization of their synergy and paradoxical “love, not war” relationship (4). Indeed, without the multifaceted involvement of many normal cells in the oncological process, the tumor could neither arise nor metastasize. This gives grounds for considering “carcinogenesis as collective rather than individual ‘guilt,' and puts the blame on the whole cellular community rather than on a single cell” (19).

Clarifying the details of this suicidal liaison can help identify principally new drugs aimed at blocking it, devoid of the side effects of the currently used cytotoxic drugs. In this way, simple and effective solutions may be found, akin to the application of soda to normalize tissue pH (69, 70) or DNase I to dissolve NETs (58, 59).

Since the structure and function of each organ are tightly interconnected, one can suggest that the blockage of the killer function may by itself help achieve the long-awaited goal of complete elimination of tumor organ. As Sidney Farber noticed: “It is not necessary, in order to make great progress in the cure of cancer, for us to have the full solution of all the problems of basic research. The history of medicine is replete with examples of cures obtained years, decades, and even centuries before the mechanism of action was understood for these cures—from vaccination, to digitalis, to aspirin” [cited by (1)].

## Author Contributions

The author confirms being the sole contributor of this work and has approved it for publication.

### Conflict of Interest Statement

The author declares that the research was conducted in the absence of any commercial or financial relationships that could be construed as a potential conflict of interest.

## Abbreviations

miRNAs: microRNAs
NETs: neutrophil extracellular traps
NETosis: neutrophil extracellular traps in peripheral vessels causing organ failure.
